# Marburg Virus Evades Interferon Responses by a Mechanism Distinct from Ebola Virus

**DOI:** 10.1371/journal.ppat.1000721

**Published:** 2010-01-15

**Authors:** Charalampos Valmas, Melanie N. Grosch, Michael Schümann, Judith Olejnik, Osvaldo Martinez, Sonja M. Best, Verena Krähling, Christopher F. Basler, Elke Mühlberger

**Affiliations:** 1 Department of Microbiology, Mount Sinai School of Medicine, New York, New York, United States of America; 2 Department of Microbiology, Boston University School of Medicine, Boston, Massachusetts, United States of America; 3 National Emerging Infectious Diseases Laboratories Institute, Boston, Massachusetts, United States of America; 4 Department of Virology, Philipps University of Marburg, Marburg, Germany; 5 Laboratory of Virology, Rocky Mountain Laboratories, National Institute of Allergy and Infectious Diseases, National Institutes of Health, Hamilton, Montana, United States of America; University of Wisconsin-Madison, United States of America

## Abstract

Previous studies have demonstrated that Marburg viruses (MARV) and Ebola viruses (EBOV) inhibit interferon (IFN)-α/β signaling but utilize different mechanisms. EBOV inhibits IFN signaling via its VP24 protein which blocks the nuclear accumulation of tyrosine phosphorylated STAT1. In contrast, MARV infection inhibits IFNα/β induced tyrosine phosphorylation of STAT1 and STAT2. MARV infection is now demonstrated to inhibit not only IFNα/β but also IFNγ-induced STAT phosphorylation and to inhibit the IFNα/β and IFNγ-induced tyrosine phosphorylation of upstream Janus (Jak) family kinases. Surprisingly, the MARV matrix protein VP40, not the MARV VP24 protein, has been identified to antagonize Jak and STAT tyrosine phosphorylation, to inhibit IFNα/β or IFNγ-induced gene expression and to inhibit the induction of an antiviral state by IFNα/β. Global loss of STAT and Jak tyrosine phosphorylation in response to both IFNα/β and IFNγ is reminiscent of the phenotype seen in Jak1-null cells. Consistent with this model, MARV infection and MARV VP40 expression also inhibit the Jak1-dependent, IL-6-induced tyrosine phosphorylation of STAT1 and STAT3. Finally, expression of MARV VP40 is able to prevent the tyrosine phosphorylation of Jak1, STAT1, STAT2 or STAT3 which occurs following over-expression of the Jak1 kinase. In contrast, MARV VP40 does not detectably inhibit the tyrosine phosphorylation of STAT2 or Tyk2 when Tyk2 is over-expressed. Mutation of the VP40 late domain, essential for efficient VP40 budding, has no detectable impact on inhibition of IFN signaling. This study shows that MARV inhibits IFN signaling by a mechanism different from that employed by the related EBOV. It identifies a novel function for the MARV VP40 protein and suggests that MARV may globally inhibit Jak1-dependent cytokine signaling.

## Author Summary

The closely related members of the filovirus family, Ebola virus (EBOV) and Marburg virus (MARV), cause severe hemorrhagic disease in humans with high fatality rates. Infected individuals exhibit dysregulated immune responses which appear to result from several factors, including virus-mediated impairment of innate immune responses. Previous studies demonstrated that both MARV and EBOV block the type I interferon-induced Jak-STAT signaling pathway. For EBOV, the viral protein VP24 mediates the inhibitory effects by interfering with the nuclear translocation of activated STAT proteins. Here, we show that MARV uses a distinct mechanism to block IFN signaling pathways. Our data revealed that MARV blocks the phosphorylation of Janus kinases and their target STAT proteins in response to type I and type II interferon and interleukin 6. Surprisingly, the observed inhibition is not achieved by the MARV VP24 protein, but by the matrix protein VP40 which also mediates viral budding. Over-expression studies indicate that MARV VP40 globally antagonizes Jak1-dependent signaling. Further, we show that a MARV VP40 mutant defective for budding retains interferon antagonist function. Our results highlight a basic difference between EBOV and MARV, define a new function for MARV VP40 and reveal new targets for the development of anti-MARV therapies.

## Introduction

Filoviruses, which include the genera *ebolavirus* (EBOV) and *marburgvirus* (MARV), are enveloped negative-strand RNA viruses that cause highly lethal hemorrhagic fever in humans and in non-human primates**.** The ability of filoviruses to counteract innate antiviral responses of the host, particularly the IFNα/β response is thought to promote uncontrolled virus replication *in vivo* and thereby contribute to development of severe disease [1]. The IFNs, which include IFNα/β and IFNγ, are antiviral cytokines. IFNα/β are members of a family of proteins that interact with the same ubiquitous receptor to trigger innate antiviral defense mechanisms and promote adaptive immunity [2]. IFNγ also triggers expression of antiviral genes, however, its major function is to modulate adaptive immune responses [3]. IFNα/β signaling results in the tyrosine phosphorylation and activation of the Janus kinases Jak1 and Tyk2. These phosphorylate STAT2 and STAT1, which in turn heterodimerize and associate with interferon regulatory factor 9 (IRF9) to form a complex that is translocated into the nucleus to activate genes involved in antiviral response (reviewed in [4]). IFNγ signaling activates Jak1 and Jak2, resulting in tyrosine phosphorylation of STAT1. This induces STAT1 homodimerization and translocation to the nucleus such that IFNγ dependent gene expression is induced (reviewed in [4]). Of note, Jak1, a kinase involved in multiple cytokine signaling pathways, is critical for both IFNα/β and IFNγ signaling. For example, in cells lacking Jak1, IFNα/β fails to trigger STAT1 or STAT2 tyrosine phosphorylation and Tyk2 tyrosine phosphorylation is greatly reduced or eliminated [5],[6]. Similarly, in cells lacking Jak1, IFNγ fails to trigger Jak1, Jak2 or STAT1 tyrosine phosphorylation [5],[7],[8].

Filovirus genomes encode seven structural proteins. Four of these proteins, the nucleoprotein (NP), the viral proteins VP35 and VP30, and the L protein are tightly associated with the RNA genome, form the nucleocapsid and mediate replication and transcription (reviewed in [9]). Besides its function as polymerase cofactor, VP35 acts as an inhibitor of antiviral pathways (see below). Two of the filovirus structural proteins are matrix proteins, VP40, the functional equivalent of the matrix (M) proteins of other non-segmented negative-stand RNA viruses, and the minor matrix protein VP24 that is unique to filoviruses. As a peripheral membrane protein VP40 is located at the inner side of the virion membrane. It is critical for viral budding and interacts with cellular proteins involved in vesicle formation to facilitate virus release [10],[11],[12],[13],[14],[15],[16],[17],[18]. The minor matrix protein VP24 is involved in nucleocapsid formation and assembly [19],[20],[21],[22],[23]. EBOV VP24 plays a crucial role in host tropism [24],[25] and is able to counteract the type I IFN response (see below). Filoviruses possess a single surface protein, the type I transmembrane glycoprotein GP that mediates attachment to target cells and virus entry. Besides EBOV VP35 and VP24, EBOV GP is the third filoviral protein known to interfere with antiviral cellular functions [26].

Among filoviruses, IFN evasion strategies have been most thoroughly explored for EBOVs. The EBOV species *Zaire ebolavirus* (ZEBOV) suppresses production of IFNα/β and inhibits cellular responses to IFNα/β and IFNγ [27],[28],[29],[30]. Inhibition of IFNα/β production appears to be mediated by the VP35 protein [31],[32], whereas cellular responses to IFNα/β and IFNγ are blocked by the EBOV VP24 protein [33],[34]. EBOV VP24 prevents the IFN-induced nuclear accumulation of tyrosine phosphorylated STAT1. This results in inhibition of IFN-induced gene expression and blocks the antiviral effects of IFNs. The inhibition of STAT1 nuclear accumulation is mediated by interaction of VP24 with NPI-1 subfamily of karyopherin α proteins that normally transport dimerized phospho-STAT1 to the nucleus [33],[34].

MARVs have a genome organization similar to EBOVs, but they are phylogenetically distinct from EBOVs [35]. Despite their similar genomic organization, morphology and the similarity of MARV versus EBOV induced disease, several biological differences between the viruses have been noted, such as differences in their transcription strategies [36], in the structure of their replication promoters [37], the use of mRNA editing to express the surface glycoprotein by EBOVs but not MARVs [38],[39] and differences in the protein requirement for nucleocapsid formation [40],[41]. In terms of the capacity of EBOV and MARV to counteract host IFN responses, microarray analyses suggest that ZEBOV and MARV each efficiently suppress host IFN responses, and each virus effectively inhibits cellular responses to exogenously added IFNα [30]. However, examination of the phosphorylation status of STAT1 following addition of IFNα to infected cells revealed an intriguing difference between ZEBOV and MARV. While ZEBOV did not inhibit the IFNα-induced tyrosine phosphorylation of STAT1, MARV infection resulted in an inhibition of both STAT1 and STAT2 tyrosine phosphorylation [30].

The present study demonstrates that MARV infection inhibits not only IFNα/β but also IFNγ and Jak1-dependent IL-6 signaling. Further, the MARV protein mediating these effects has been identified. We show that expression of the MARV matrix protein VP40 is sufficient to block IFN and IL-6 signaling pathways. Experiments in which either Jak1 or Tyk2 are over-expressed suggest that MARV VP40 targets Jak1 function. These observations identify an important difference in the biology of MARV and EBOVs, identify a novel function for a negative-strand RNA virus matrix protein and suggest that MARV may inhibit multiple Jak1-dependent cytokine signaling pathways.

## Results

### MARV infection prevents IFN-mediated phosphorylation and nuclear translocation of STAT proteins

**Figure 1:**
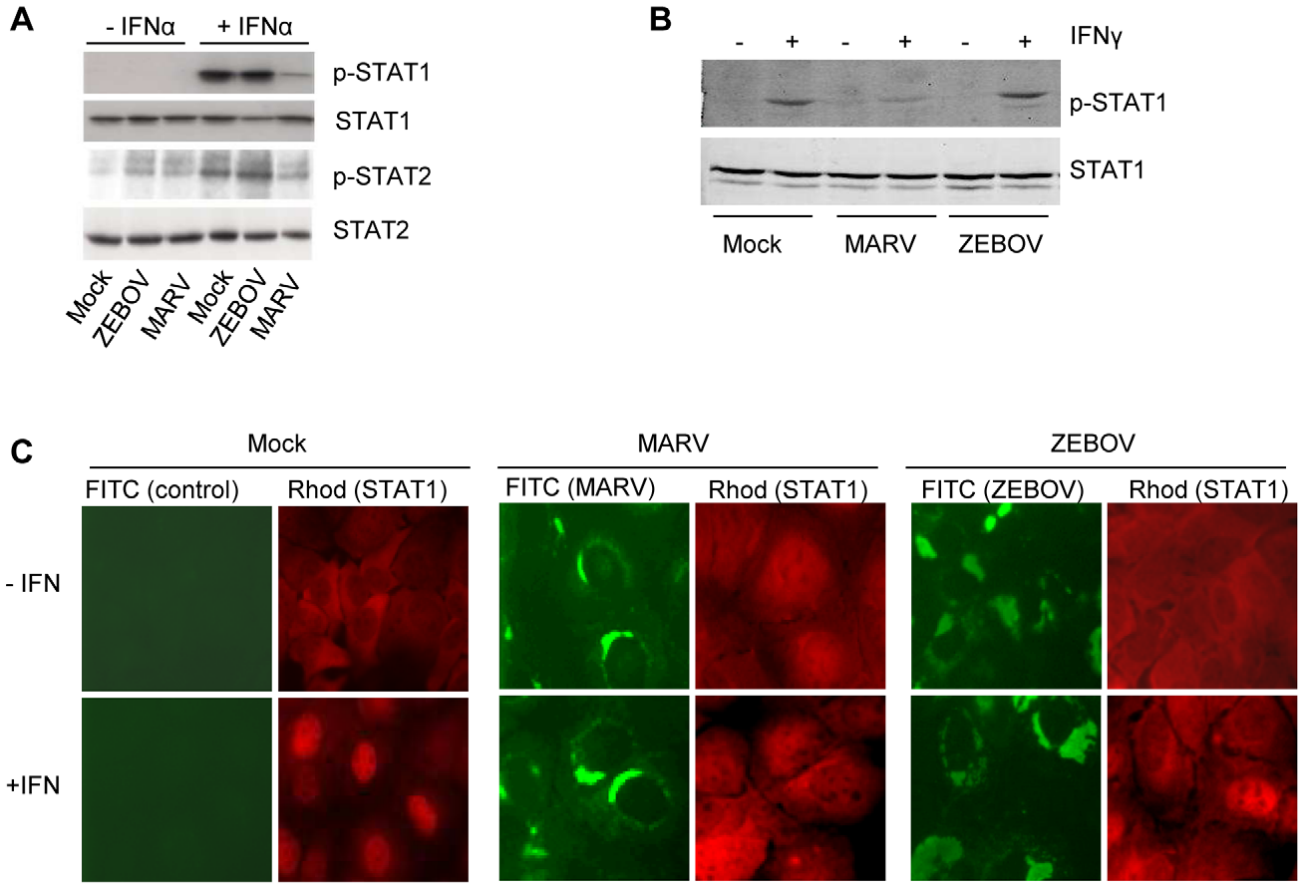
MARV infection prevents IFN-mediated phosphorylation and nuclear translocation of STAT proteins. Huh-7 cells were infected with MARV or ZEBOV at an MOI of 5 or mock-infected (Mock). At 24 h p.i., cells were either treated with 100 IU/ml of IFNα-2b (A) or 10 IU/ml of IFNγ (B) for 30 min where indicated. Cell lysates were analyzed by western blotting using antibodies directed against total and phosphorylated forms of STAT1 (A and B) or STAT2 (A). (C) Huh-7 cells were infected with MARV or ZEBOV at an MOI of 5 or left uninfected. At 24 h p.i., the cells were stimulated with 1000 IU/ml of IFNα-2b for 45 min, fixed with 4% paraformaldehyde, and stained with antibody directed against STAT1, MARV, or ZEBOV as indicated.

Previous studies demonstrated that tyrosine phosphorylation of STAT1 and STAT2 is strongly reduced in MARV- but not in ZEBOV-infected Huh-7 cells treated with IFNα [30]. To confirm this observation and to determine whether MARV inhibition extends to other Jak-STAT signaling pathways, the impact of MARV infection on IFNα-induced STAT1 and STAT2 phosphorylation and on IFNγ-induced STAT1 phosphorylation was compared. As reported, MARV but not EBOV inhibited phosphorylation of endogenous STAT1 and STAT2 induced by IFNα (Fig. 1A). MARV also inhibited IFNγ-induced STAT1 phosphorylation, whereas EBOV did not (Fig. 1B). For these studies, immunofluorescence analyses were performed in parallel to confirm that more than 95% of cells were infected with either virus (data not shown). These data show that MARV not only blocks type I but also type II IFN signaling by interfering with an early step of the Jak-STAT signaling cascade.

Since previous studies indicated that the nuclear translocation of phosphorylated STAT1 is inhibited in EBOV-infected cells [33],[34], we examined the cellular localization of STAT1 in MARV-infected cells by immunofluorescence (Fig. 1C). As expected, STAT1 was translocated into the nucleus in non-infected cells treated with IFNα (left panels, red staining), whereas IFNα-induced translocation was inhibited in ZEBOV-infected cells (right panels, infected cells shown in green). Please note that a single non-infected cell in the ZEBOV infection panel showed nuclear accumulation of STAT1. Nuclear translocation of STAT1 was also blocked in MARV-infected cells treated with IFNα (middle panels).

Taken together, these results highlight a fundamental difference in the mechanisms by which MARV and EBOV counteract innate immune responses.

### IFNα-induced tyrosine phosphorylation of Janus kinases is inhibited in MARV-infected cells

**Figure 2:**
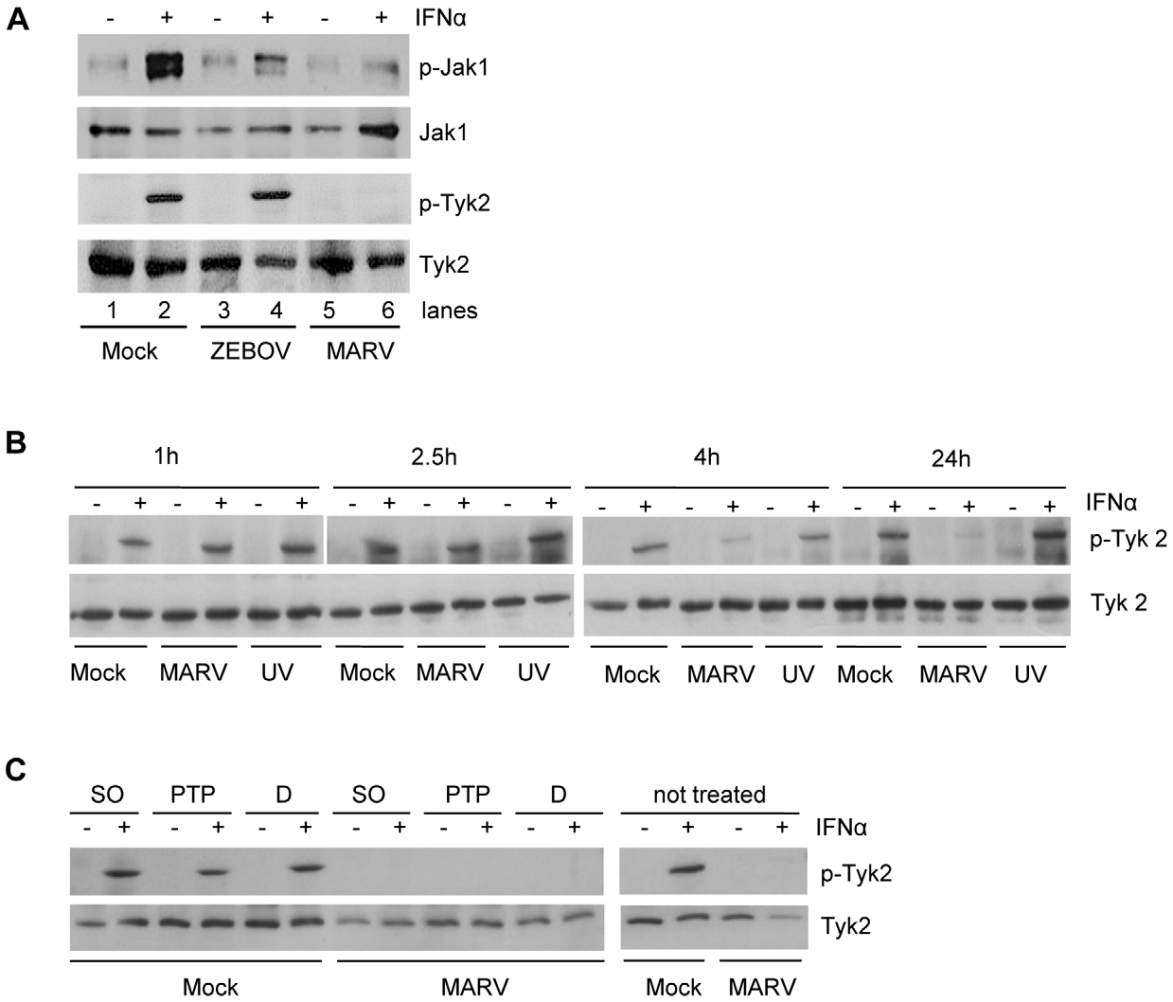
IFNα-induced tyrosine phosphorylation of Janus kinases is inhibited in MARV- but not in EBOV-infected cells. This inhibition occurs early in infection and is insensitive to PTP inhibitors. (A) Huh-7 cells were infected with MARV or ZEBOV at an MOI of 5 or left uninfected. At 24 h p.i., cells were treated with 1000 IU/ml of IFNα for 20 min where indicated. Cell lysates were analyzed by western blotting using antibodies directed against total protein and phosphorylated forms of Jak1 and Tyk2. (B) Huh-7 cells were infected with MARV or UV-inactivated MARV at an MOI of 5 or left uninfected. 20 min before lysis (24 h p.i.), cells were treated with 2000 IU/ml of IFNα and harvested at the indicated time points. Cell lysates were analyzed by western blotting using antibodies directed against total protein and phosphorylated forms of Tyk2. (C) Huh-7 cells were infected with MARV at an MOI of 5. The cells were treated with DMSO (D) or the phosphatase inhibitors sodium orthovanadate (SO) or PTP1B inhibitor (PTP) prior to IFN treatment (24 h p.i., 2000 IU/ml IFNα for 20 min). Cell lysates were analyzed by western blotting using antibodies directed against total protein and phosphorylated forms of Tyk2. Note that the cuts in the films excised samples irrelevant to this study.

Since our data suggested that MARV infection leads to the inhibition of IFN-induced STAT phosphorylation, we next sought to determine the activation status of Jak1 and Tyk2, the Janus kinases involved in IFNα-induced phosphorylation of STAT proteins. Huh-7 cells were infected with MARV or ZEBOV, treated with IFNα and the phosphorylation state of endogenous Jak1 and Tyk2 was analyzed by western blot analysis. As shown in Figure 2A, both kinases were phosphorylated in ZEBOV-infected cells in response to IFNα, although phosphorylation of Jak1 was less pronounced compared to non-infected cells (Fig. 2A, compare lane 2 and 4). However, only background levels of Jak1 phosphorylation were detectable and Tyk2 phosphorylation was completely blocked in MARV-infected cells treated with IFNα (Fig. 2A, lane 6). From this we concluded that the inhibition of the Jak-STAT-signaling pathway by MARV takes place upstream of Jak phosphorylation or directly at the Janus kinases.

As part of the innate immune response, the Jak-STAT signaling cascade acts as a first line of defense to prevent viral infections. Therefore, we determined at which time point of the MARV replication cycle the observed inhibition of Jak activation occurs. Further, we asked whether live virus and viral replication are needed to antagonize IFN signaling. Huh-7 cells were infected with live MARV or UV-inactivated MARV, treated with IFNα, harvested at different time points post-infection (p.i.) and subjected to western blot analysis to determine the phosphorylation state of Tyk2. While Tyk2 was still efficiently phosphorylated in MARV-infected and IFN-treated cells at 1 hour and 2.5 hours p.i., respectively, near complete inhibition of Tyk2 phosphorylation was achieved at 4 hours p.i. (Fig. 2B). A single MARV replication cycle takes approximately 21 hours in Vero E6 cells [42]. Thus, it can be concluded that the observed antagonistic effect occurs early in infection. Additionally, since MARV infection did not lead to the inhibition of Tyk2 phosphorylation at time points earlier than 4 hours p.i., it is assumed that binding of MARV to its receptor does not trigger its IFN antagonist function. Interestingly, infection of cells with UV-inactivated MARV prior to IFNα treatment did not lead to the inhibition of Tyk2 phosphorylation (Fig. 2B), supporting the assumption that receptor binding does not play a role in the MARV-specific inhibition of the IFN signaling cascades. In addition, these data indicate that intracellular virus replication is required for the observed antagonistic effects.

To examine whether MARV indirectly inhibits Jak1 phosphorylation via protein tyrosine phosphatases (PTPs), we treated MARV-infected and IFN-treated cells with different PTP inhibitors prior to IFNα stimulation. Besides an inhibitor against PTP1B, which specifically dephosphorylates Tyk2 and Jak2 [43], we tested the broad acting phosphatase inhibitor sodium orthovanadate. Our results show that even in the presence of PTP inhibitors Tyk2 phosphorylation was inhibited in MARV-infected cells (Fig. 2C), suggesting that the observed inhibitory effects do not depend on active cellular PTPs.

### MARV matrix protein VP40 acts as an IFN antagonist

**Figure 3:**
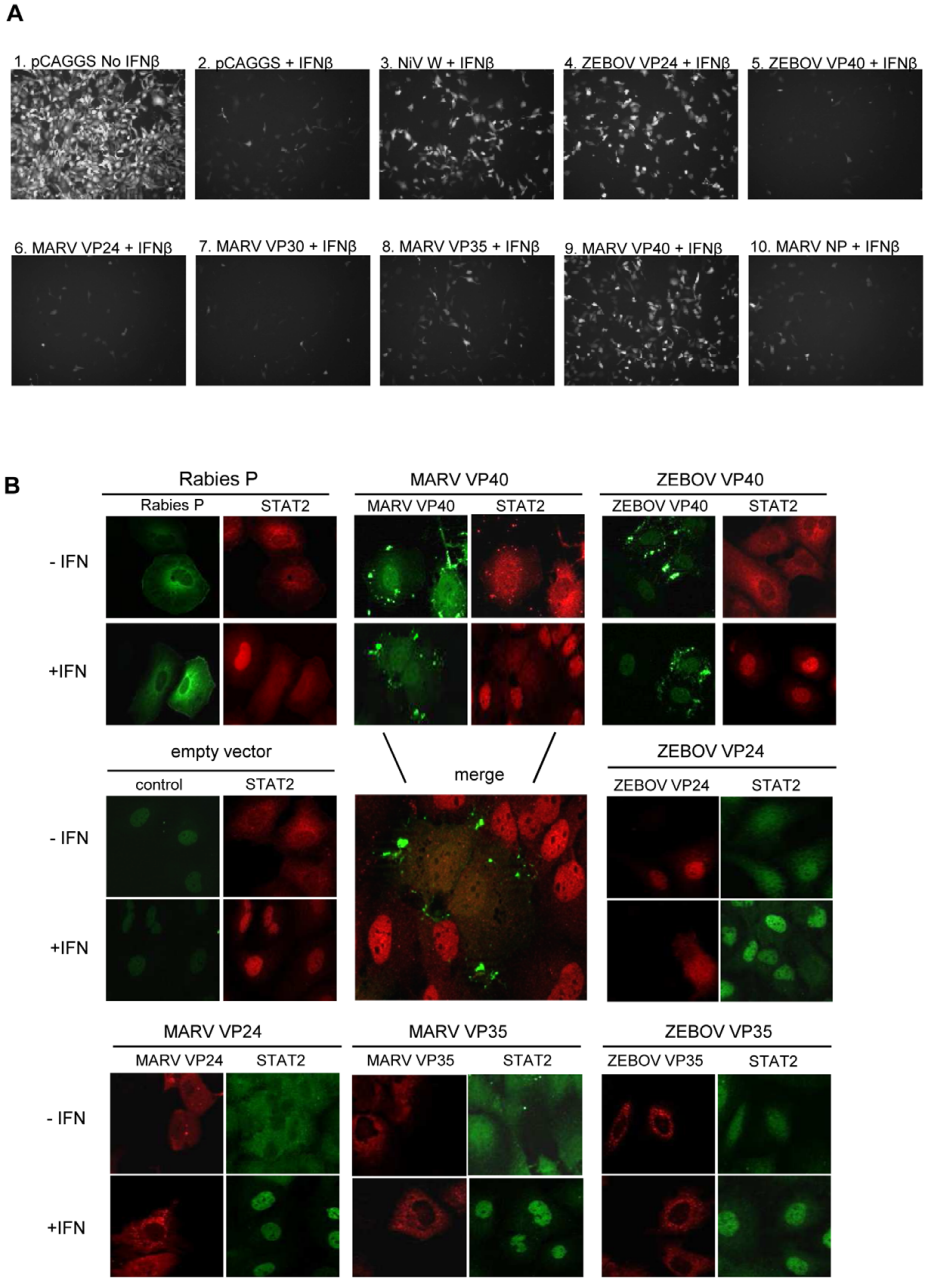
MARV VP40 acts as an IFN antagonist. (A) Vero cells were transfected with 1 µg empty vector (pCAGGS) or the indicated expression plasmids. 24 h post transfection (p.t.) cells were treated with 1000 IU/ml of IFNβ for 24 h and infected with NDV GFP. At 16 h p.i., green fluorescence (indicating viral replication) was visualized and photographed with a fluorescence microscope. (B) Huh-7 cells were transfected with 2 µg of the indicated plasmids expressing the indicated viral proteins from rabies virus, ZEBOV and MARV. At 24 h p.t., the cells were stimulated with 2000 IU/ml of IFNα-2b for 45 min, fixed with 4% paraformaldehyde, and stained with anti-STAT2 antibody and antibodies to detect viral proteins.

To identify the viral protein mediating the antagonistic effects observed in MARV-infected cells, individual EBOV or MARV proteins were assessed for their capacity to counteract the antiviral effects of IFNβ (Fig. 3A). Vero cells were transfected with expression plasmids; one day post-transfection the cells were either mock-treated or treated overnight with IFNβ, and the cells were then infected with a Newcastle disease virus that expresses GFP (NDV-GFP). Since NDV is IFN-sensitive, GFP expression in these cells provides a measure of virus replication, and suppression of GFP expression provides a read-out for the antiviral effects of IFNβ. While empty vector (pCAGGS)-transfected, mock-treated cells permitted NDV-GFP replication, IFNβ-treated, empty vector-transfected cells, in contrast, greatly suppressed GFP expression (Fig. 3A, panels 1 and 2). As previously described, expression of Nipah virus W protein, or ZEBOV VP24, known inhibitors of IFN signaling, rescued replication of NDV-GFP in IFNβ-treated cells [33],[44] (Fig. 3A, panel 3 and 4). Surprisingly, MARV VP24 did not detectably counteract the antiviral effects of IFNβ (Fig. 3A, panel 6). In fact, the only MARV protein tested that clearly permitted NDV-GFP replication in IFNβ-treated cells was the major matrix protein VP40 (Fig. 3A, panel 9). In contrast, the homologous ZEBOV protein, ZEBOV VP40, did not support NDV-GFP replication (Fig. 3A, panel 5).

To confirm the finding that MARV VP40 antagonizes IFN signaling, we analyzed the intracellular distribution of STAT2 in cells transiently expressing MARV or EBOV proteins VP35, VP24, or VP40 (Fig. 3B). Since it has been shown by Brzozka et al. [45] that rabies virus phosphoprotein (P) efficiently blocks the nuclear translocation of STAT2 into the nucleus, P was used as a positive control (Fig. 3B). Cells transfected with empty vector served as a negative control. While expression of either MARV VP40 or ZEBOV VP24 led to a significant inhibition of STAT2 accumulation in the nucleus, none of the other tested filoviral proteins including ZEBOV VP40 and MARV VP24 was able to inhibit nuclear translocation of STAT2 in response to IFNα (Fig. 3B). From this, we concluded that MARV VP40 is the viral protein interfering with IFN signaling.

### MARV VP40 inhibits type I and type II IFN-induced STAT and Jak activation

**Figure 4:**
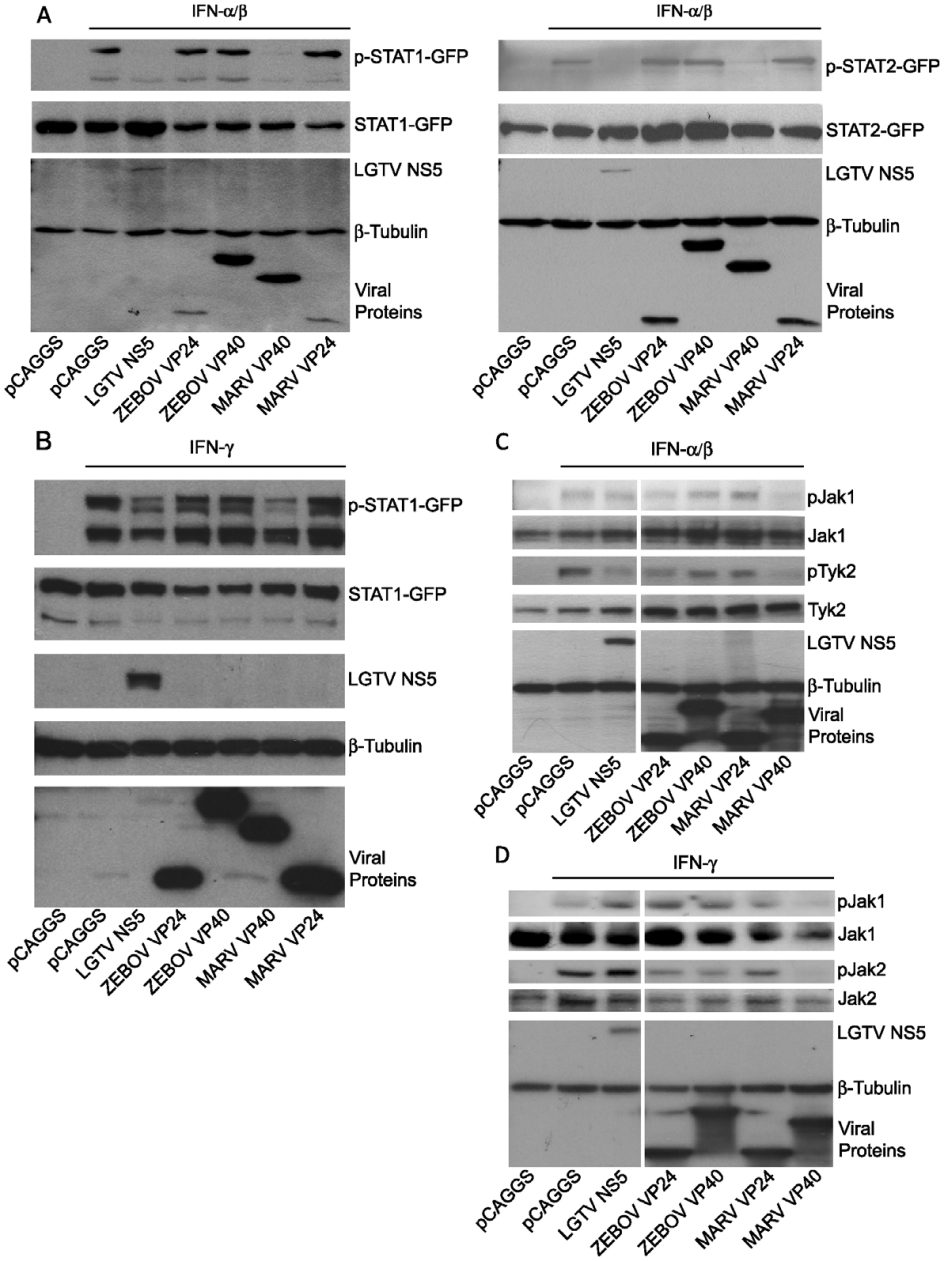
MARV VP40 inhibits IFN-induced STAT and Jak phosphorylation. (A) STAT1 or STAT2 (1 µg) fused to a C-terminal GFP was co-expressed in Huh-7 cells with 2 µg of the indicated expression plasmids, treated with 1000 IU/ml of universal IFNα/β for 30 min. Cells were lysed and assayed by western blot for tyrosine phosphorylated STAT1 (p-STAT1-GFP), STAT2 (p-STAT2-GFP), as well as for total expression levels of over-expressed proteins. (B) Huh-7 cells were transfected with 2 µg of the indicated expression plasmids and 1 µg of a plasmid expressing STAT1 fused to GFP at the C-terminus (STAT1-GFP). 24h p.t., cells were treated with 1000 IU/ml of IFNγ for 30 min and lysed. Lysates were analyzed by western blot for phosphorylation of STAT1 and total levels of STAT1 as well as for expression of the tagged proteins. (C and D) 293T cells were transfected with 2 µg of the indicated expression plasmids, treated with 1000 IU/ml of IFNα/β (C) or IFNγ (D) for 30 min, lysed and subjected to western blot analysis for detection of phosphorylated and total Jak1 (C and D), Tyk2 (C) or Jak2 (D). Note that the cuts in the films excised samples irrelevant to this study. All the presented data for a given protein is from the same gel and the same exposure.

Our results obtained with infected cells clearly show that MARV infection leads to the inhibition of STAT and Jak phosphorylation, whereas ZEBOV infection does not. To assess the impact of MARV VP40 on IFN-induced signaling in the absence of other viral proteins, STAT1-GFP or STAT2-GFP were co-transfected into Huh-7 cells with empty vector or with plasmids expressing ZEBOV VP40, ZEBOV VP24, MARV VP40 or MARV VP24. The phosphorylation state of the STAT proteins in response to IFNα/β (Fig. 4A) and IFNγ (Fig. 4B) was examined by western blot analysis. Expression of the Langat virus NS5 protein (LGTV NS5), a protein previously demonstrated to inhibit STAT1 and STAT2 tyrosine phosphorylation [46] served as a control. Following addition of IFNα/β to transfected Huh-7 cells, MARV VP40 inhibited the IFNα/β -induced tyrosine phosphorylation of either STAT1-GFP (Fig. 4A, left panel) or STAT2-GFP (Fig. 4A, right panel). In contrast, the ZEBOV VP40, ZEBOV VP24 and MARV VP24 proteins failed to inhibit STAT1 or STAT2 tyrosine phosphorylation (Fig. 4A). Relative to empty vector-transfected cells, LGTV NS5 reduced the IFNγ-induced phosphorylation of STAT1-GFP (Fig. 4B), but ZEBOV VP24, MARV VP24 and ZEBOV VP40 failed to inhibit STAT1 phosphorylation. In contrast, MARV VP40 expression led to a substantial reduction in IFNγ-induced STAT1 tyrosine phosphorylation (Fig. 4B).

Next, we analyzed the impact of MARV VP40 on the phosphorylation of Janus kinases in cells treated with IFNα/β or IFNγ. 293T cells were transfected with empty vector or plasmids that express LGTV NS5, ZEBOV VP24, ZEBOV VP40, MARV VP24 or MARV VP40, treated with IFNα/β and analyzed for phosphorylation of endogenous Jak1 and Tyk2. MARV VP40 inhibited the IFNα/β-induced tyrosine phosphorylation of both kinases (Fig. 4C). Interestingly, none of the other expressed proteins including LGTV NS5 detectably blocked Jak1 phosphorylation. Although Tyk2 phosphorylation was also reduced by LGTV NS5 and to a lesser extent by ZEBOV VP24, this reduction was less pronounced compared to cells expressing MARV VP40 (Fig. 4C). Similar results were obtained in cells treated with IFNγ. Inhibition of Jak1 and Jak2 phosphorylation in response to IFNγ treatment was only observed in cells expressing MARV VP40 (Fig. 4D). Taken together, these results clearly confirm that MARV not only uses a different mechanism than EBOV to block IFN signaling, but an alternate viral protein carries out this function.

### MARV VP40 inhibits ISRE- and GAS-induced gene expression

**Figure 5:**
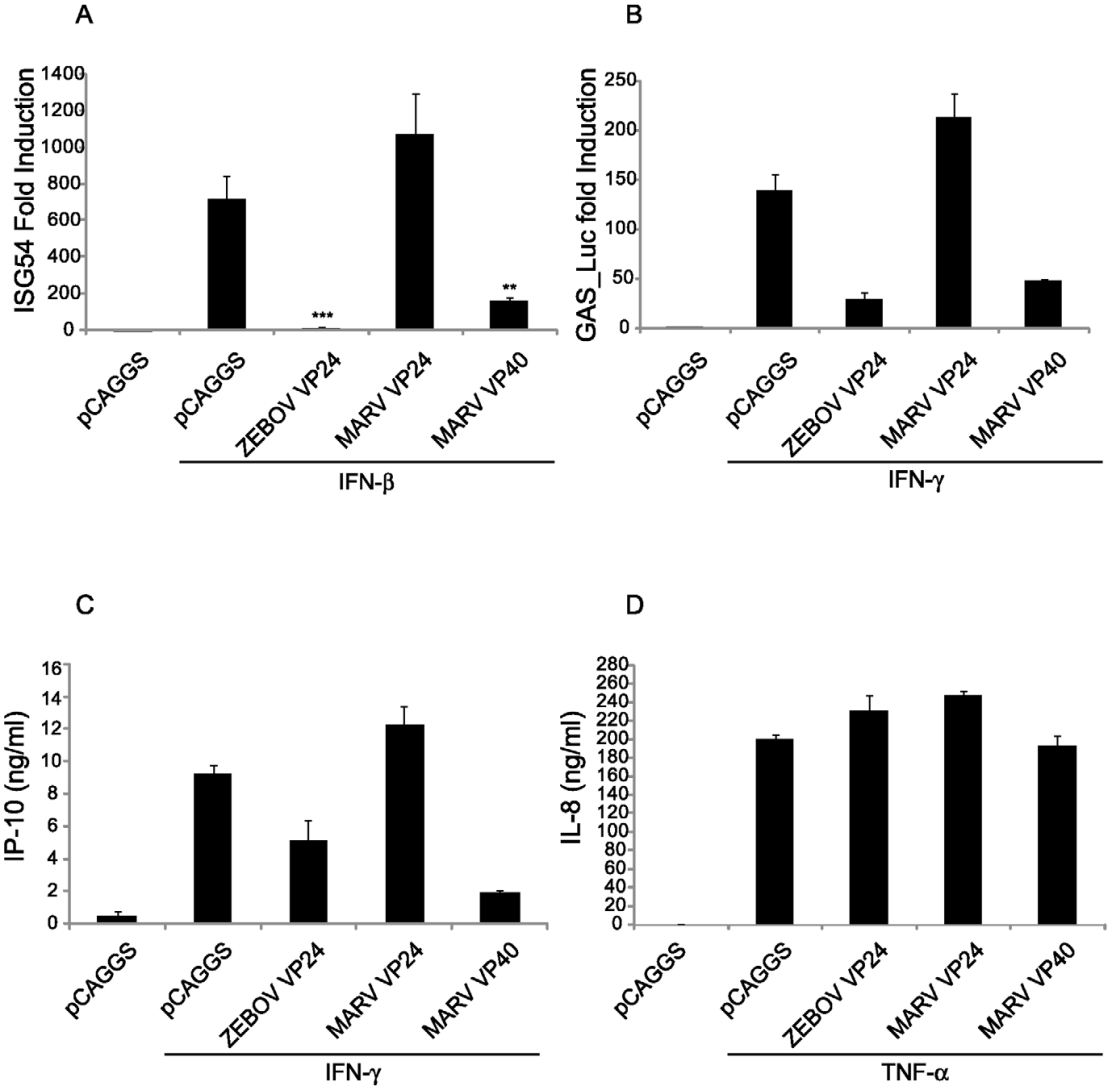
MARV VP40 inhibits ISRE- and GAS-induced gene expression. (A) MARV VP40 inhibits type I IFN-induced gene expression. 293T cells were co-transfected with a construct expressing the CAT gene driven by an ISG54 promoter along with a constitutively expressed *Renilla* luciferase gene and 1 µg of plasmids that express the indicated viral proteins and 24h.p.t. treated with 1000 IU/ml IFNα/β for 18 h and assayed for CAT and luciferase activities. The IFN-induced CAT activity was normalized to *Renilla* luciferase activity. Presented is the mean fold induction of 3 experiments compared to the untreated negative control, error bars represent the standard deviations and the asterisks the p-values (**p-val = 0.016; ***p = 0.0006). Lysates were analyzed for viral protein expression (data not shown). (B) MARV VP40 inhibits IFNγ dependent gene expression. Huh-7 cells were co-transfected with the IFNγ inducible firefly luciferase reporter plasmid pGAS-Luc along with a constitutively expressed *Renilla* luciferase gene and 0.6 µg of the indicated expression plasmids. Cells were treated with a 1000 IU/ml of IFNγ for 18 h and assayed for dual luciferase activities. The IFN-induced firefly luciferase activity was normalized to *Renilla* luciferase activity. The bars represent the mean fold induction of 3 experiments compared to the untreated negative control, and the error bars represent the standard deviations. (C) MARV VP40 inhibits the IFNγ dependent IP-10 production. 2×10^5^ HUVECs were transfected with 2 µg of the indicated expression plasmids. Cells were treated with 100 IU/ml IFNγ for 24 h; and supernatants were collected, cleared by centrifugation and analyzed by ELISA for IP-10. (D) MARV VP40 does not inhibit TNFα-induced IL-8 production. HUVECs were transfected as in (C) and treated with 50 ng/ml of TNFα for 24 h. Supernatants were collected, cleared by centrifugation and analyzed by ELISA for IL-8 concentrations.

To address the functional significance of the observed inhibition, the impact of MARV VP40 on IFNβ and IFNγ-induced transcription was assessed by reporter gene assay (Fig. 5). Two reporter constructs were used. One, activated by IFNα/β, possesses an ISG54 promoter and contains an interferon stimulated response element (ISRE). The second, activated by IFNγ, possesses three gamma activated sequence (GAS) elements. 293T cells were transfected with either reporter plus expression plasmids for MARV VP40 or, as controls, MARV VP24 and ZEBOV VP24. To control for non-specific or cytotoxic effects of the viral proteins, the results of these assays were normalized to a co-transfected constitutively-expressed *Renilla* luciferase reporter plasmid. MARV VP40 and ZEBOV VP24 inhibited ISG54 promoter activation, whereas MARV VP24 failed to inhibit its activation (Fig. 5A). Similarly, MARV VP40 inhibited IFNγ-induced gene expression, consistent with its capacity to block IFNγ activation of STAT1. As previously described, ZEBOV VP24 inhibited IFNγ-induced gene expression [33], but MARV VP24 did not inhibit gene expression in this assay (Fig. 5B).

The impact of MARV VP40 upon IFNγ-induced production of the 10 kDa interferon-gamma-induced protein (IP-10), an immune cell chemoattractant protein secreted by several cell types in response to IFNγ, was also assessed. Human umbilical vein endothelial cells (HUVECs) were transfected with the indicated expression plasmids, treated with IFNγ, and cell supernatants were tested for the presence of IP-10 by ELISA. MARV VP40 and, to a lesser extent, ZEBOV VP24 inhibited IP-10 expression, whereas MARV VP24 did not (Fig. 5C). To assess the specificity of this effect and exclude cell death or disruption of membrane signaling components, a similar assay was performed testing the impact of viral protein expression on TNFα-induced secretion of IL-8 which is NF-κB-mediated [47],[48]. None of the expressed proteins, including MARV VP40, detectably affected IL-8 production (Fig. 5D). Therefore the impact of MARV VP40 seems to be specific for Jak-STAT signaling and does not extend to the induction of NF-κB-mediated signaling [47].

### MARV infection and expression of MARV VP40 inhibit IL-6-induced STAT1 and STAT3 phosphorylation

**Figure 6:**
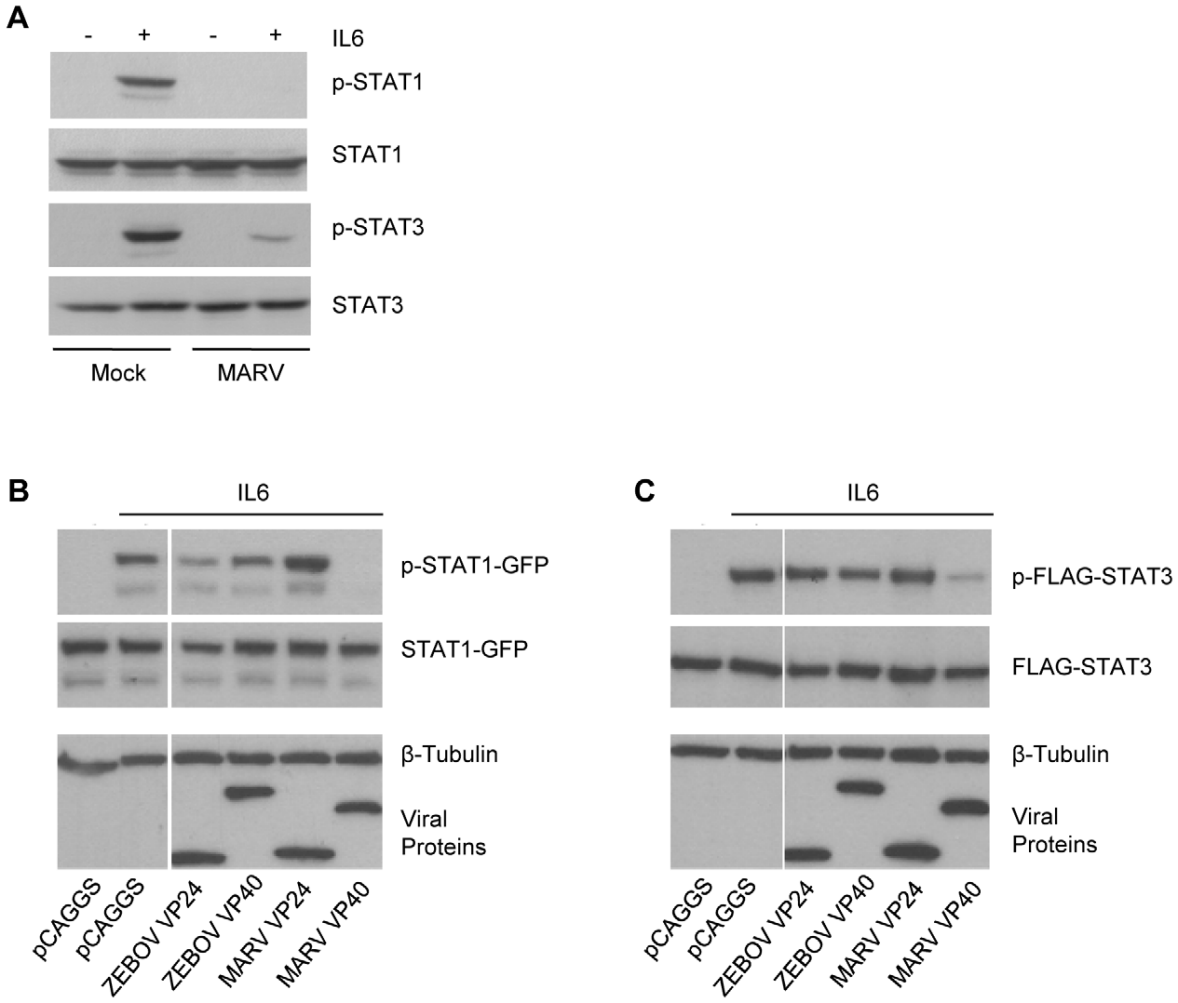
MARV inhibits IL-6 signaling. (A) Huh-7 cells were infected with MARV at an MOI of 5 or left uninfected. At 24 h p.i., cells were treated with 50 ng/ml IL-6 for 30 min where indicated. Cell lysates were analyzed by western blotting using antibodies directed against total protein and phosphorylated forms of STAT1 and STAT3. (B and C) Huh-7 cells were co-transfected with 2 µg of the indicated viral protein expression plasmids and either 1 µg of plasmids encoding STAT1-GFP (B) or FLAG-STAT3 (C). The samples were analyzed as described in (A). Note that for panels B and C, the cuts in the films excised samples irrelevant to this study. All the presented data for a given protein is from the same gel and the same exposure.

Interestingly, our observations are reminiscent of the phenotype seen in Jak1-deficient cells, where the absence of Jak1 results in loss of Jak1, Tyk2, STAT1 and STAT2 tyrosine phosphorylation in response to IFNα/β and loss of Jak1, Jak2 and STAT1 tyrosine phosphorylation in response to IFNγ [5],[7],[8]. To examine whether the observed inhibitory effect of MARV on IFN signaling extends to other, non-IFN, Jak-STAT signaling pathways, we next analyzed the IL-6-induced activation of STAT1 and STAT3 in MARV-infected cells and cells expressing VP40. IL-6 was chosen because, in Jak1-deficient cells, IL-6-induced STAT1 phosphorylation was absent, and STAT3 phosphorylation was greatly reduced [7]. Huh-7 cells were infected with MARV, treated with IL-6 at 24 hours p.i. and cell lysates were subjected to western blot analysis. As shown in Figure 6A, phosphorylation of endogenous STAT1 was not detectable and STAT3 phosphorylation was strongly diminished in MARV-infected, IL-6 treated cells, reflecting the phenotype of Jak1-deficient cells [7]. Similar results were obtained with transfected Huh-7 cells. MARV VP40 inhibited the IL-6 induced tyrosine phosphorylation of STAT1-GFP to undetectable levels, and FLAG-STAT3 tyrosine phosphorylation was highly reduced (Fig. 6B and C). In contrast, ZEBOV VP40, ZEBOV VP24 and MARV VP24 did not inhibit phosphorylation of either STAT1 or STAT3 (Fig. 6B and C).

### MARV VP40 inhibits phosphorylation of over-expressed Jak1

**Figure 7:**
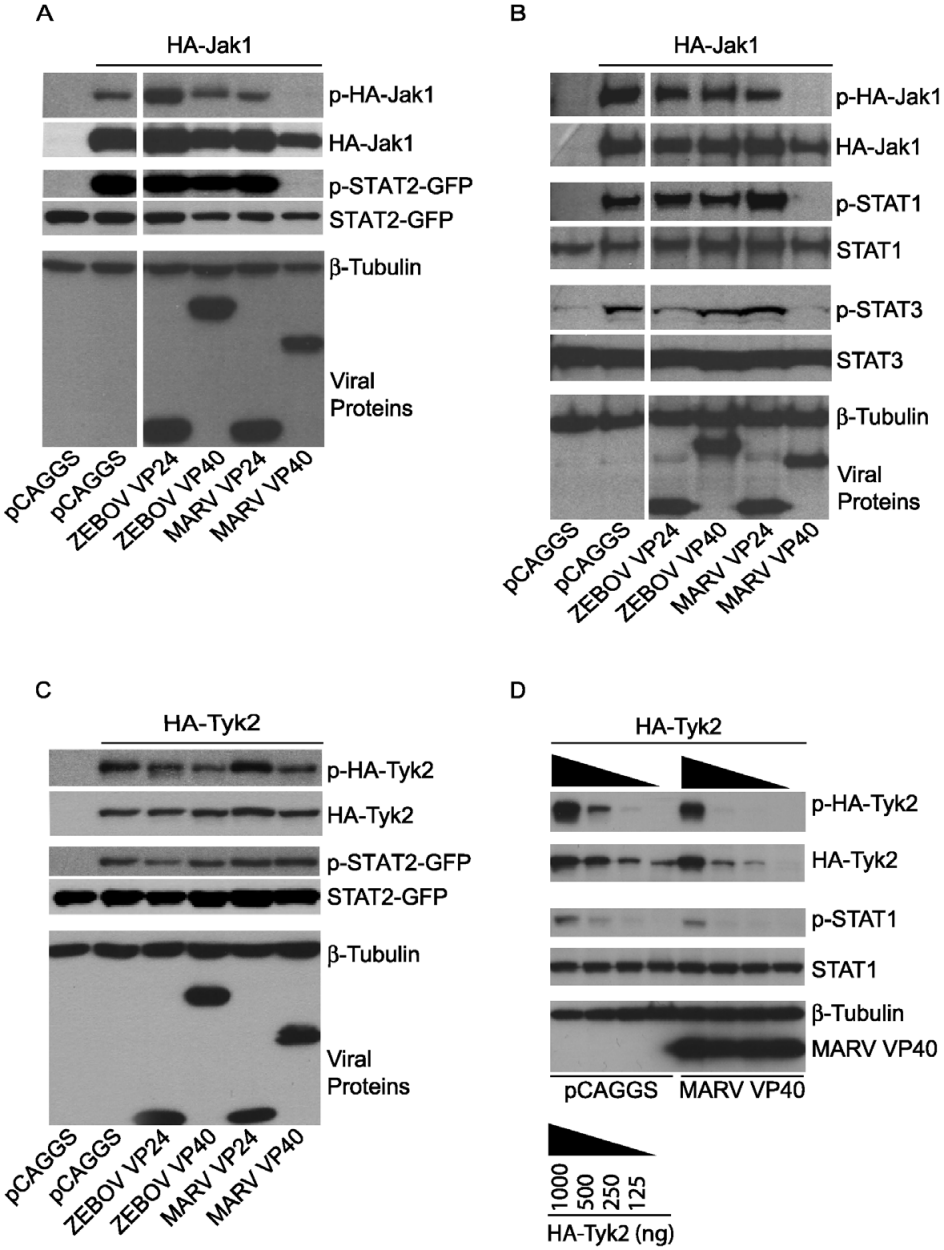
Jak1 phosphorylation is inhibited by MARV VP40. (A and C) 1 µg of HA-tagged human Jak1 (A) or Tyk2 (C) expression plasmid was transfected into Huh-7 cells along with 2 µg of empty vector (pCAGGS) or the indicated viral protein expression plasmids and 0.5 µg plasmid encoding STAT2 fused to GFP. Cells were lysed and subjected to western blot analysis using total and phospho-specific antibodies against Jak1 (A), Tyk2 (C), and STAT2 or GFP. Anti-β-tubulin was used as a loading control and anti-Flag to detect expression of viral proteins. (B) 1 µg of human Jak1 plasmid was transfected along with 2 µg of empty vector (pCAGGS) or the indicated viral protein expression plasmids. Cells were lysed, subjected to SDS-PAGE and analyzed with total and phospho-specific antibodies against Jak1, STAT1 and STAT3. Anti-β-tubulin was used as a loading control and anti-Flag to detect expression of viral proteins. (D) Two-fold dilutions of HA-Tyk2 expression plasmid starting at 1 µg were transfected in Huh-7 cells with either 2 µg of empty plasmid (pCAGGS) or 2 µg of expression plasmid for MARV VP40. Levels of phospho-Tyk2 and phospho-STAT1 as well as total Tyk2 and STAT1 were assayed by western blot. Anti-β-tubulin and anti-Flag antibodies were used as indicated. Note that for panels A and B, the cuts in the films excised samples irrelevant to this study. All the presented data for a given protein is from the same gel and the same exposure.

To further assess the capacity of MARV VP40 to target the function of Jak1, MARV VP40, ZEBOV VP40, MARV VP24 or ZEBOV VP24 were co-transfected with expression plasmids for STAT2-GFP and either HA-tagged Jak1 or HA-tagged Tyk2. Over-expression of Janus kinases leads to their tyrosine phosphorylation [49] and to the phosphorylation of STAT proteins (Fig. 7). First, we determined the phosphorylation state of HA-Jak1 and STAT2-GFP in transfected cells by western blot analysis (Fig. 7A). While MARV VP40 completely inhibited the phosphorylation of over-expressed HA-Jak1 and consequently, the phosphorylation of STAT2-GFP, ZEBOV VP40, ZEBOV VP24 and MARV VP24 did not show any inhibitory effect (Fig. 7A). Extending this observation, HA-Jak1 over-expression also led to tyrosine phosphorylation of endogenous STAT1 and STAT3, and this was inhibited by MARV VP40 but not by the other tested viral proteins (Fig. 7B). In contrast, none of the expressed filovirus proteins, including MARV VP40, detectably inhibited tyrosine phosphorylation of over-expressed HA-Tyk2 or Tyk2-induced STAT2-GFP phosphorylation (Fig. 7C). Further titration of HA-Tyk2 expression was performed, and phosphorylation of endogenous STAT1 was monitored (Fig. 7D). Two-fold dilutions of HA-Tyk2 plasmid were transfected with either empty vector or MARV VP40 plasmid. When 500 ng of Tyk2 plasmid was transfected, less phospho-Tyk2 was detected in the MARV VP40-expressing cells than in cells receiving empty vector. Similarly, levels of phosphorylated endogenous STAT1 were decreased in the presence of MARV VP40. However, the total levels of HA-Tyk2 were also decreased in the presence of MARV VP40 in these samples (Fig. 7D). Therefore the bands were quantified by densitometry and the ratio of phosphorylated Tyk2 to total Tyk2 was calculated for each sample. In all samples the ratios were in the range of 0.85 to 1.05, suggesting that the decreased levels of phospho-Tyk2 were due to reduced total levels of Tyk2 (data not shown). These data support a model where MARV VP40 targets Jak1 function but do not completely exclude the possibility that MARV VP40 has a modest capacity to inhibit Tyk2.

### MARV VP40 inhibition of Jak1-dependent signaling does not require an intact late domain

**Figure 8:**
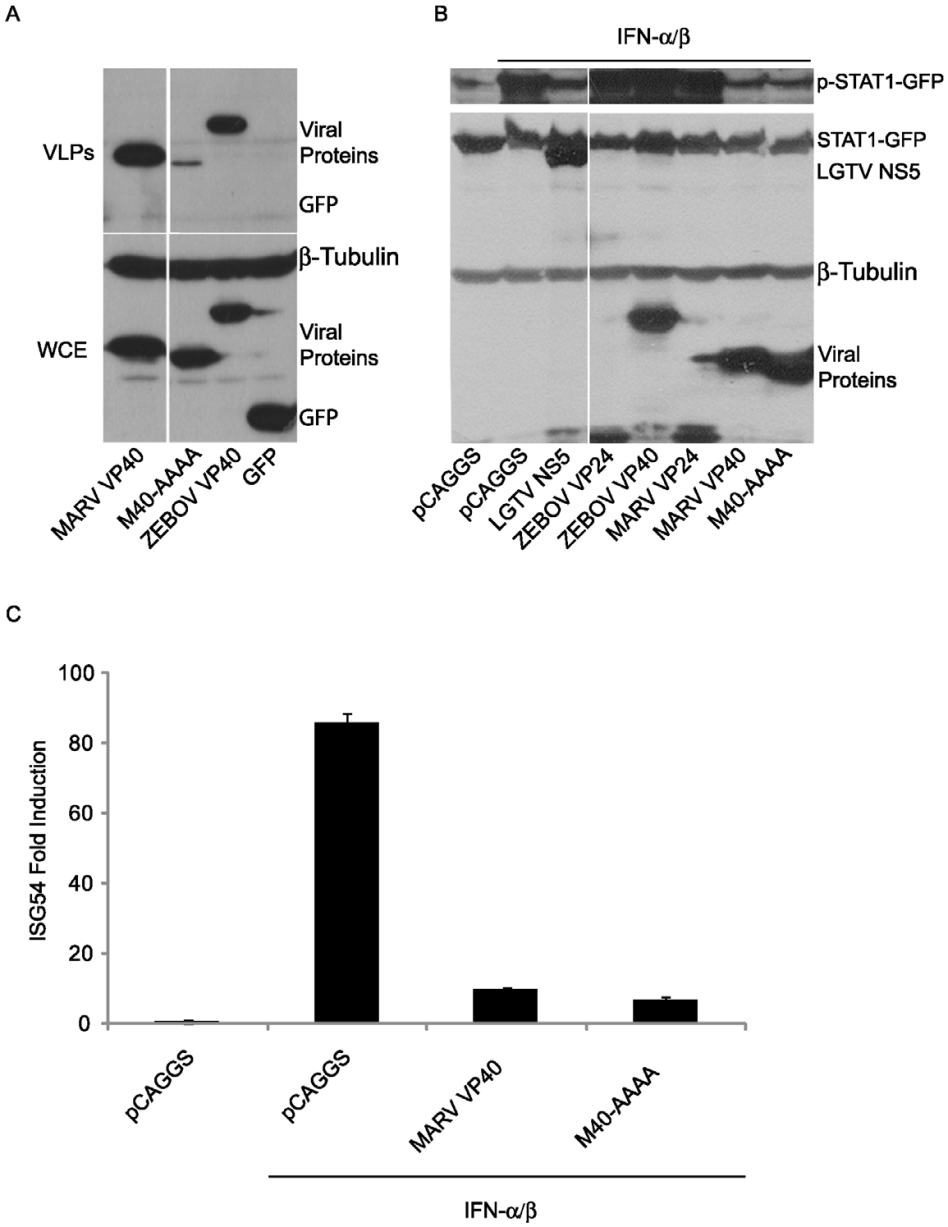
MARV VP40 inhibition of IFN signaling does not require an intact late domain. (A) M40-AAAA buds less efficiently than wild type MARV VP40. A MARV VP40 late domain mutant (M40-AAAA) was made using site directed mutagenesis. M40-AAAA, wild type MARV VP40, ZEBOV VP40 or GFP (2 µg) was expressed in 293T cells. 48 h later, supernatants were harvested and virus-like particles (VLPs) were purified through a sucrose cushion. Cells were lysed and examined together with the VLPs for protein expression levels (WCE: whole cell extract). (B) MARV VP40 does not require the late domain to inhibit the phosphorylation of STAT1. Huh-7 cells were transfected with 1 µg STAT1-GFP expression plasmid along with 2 µg plasmid DNA encoding Flag-tagged versions of viral proteins from LGTV, ZEBOV and MARV or the late domain mutant M40-AAAA and treated with 1000 IU/ml of IFNα/β for 30 min. Lysates were analyzed for phosphorylation of STAT1 and for total expression levels of all over-expressed proteins. β-tubulin expression was assessed as indicated. All the presented data for a given protein is from the same gel and the same exposure. (C) MARV VP40 does not need the late domain to inhibit the IFNα/β-dependent induction of ISG54-Luc. 293T cells were co-transfected with a construct expressing the luciferase gene under control of an ISG54 promoter along with a constitutively expressed *Renilla* luciferase reporter gene and 1 µg empty vector (pCAGGS) or expression plasmids expressing wild-type MARV VP40 or M40-AAAA. Cells were treated with 1000 IU/ml IFNα/β for 18 h and assayed for firefly and *Renilla* luciferase activities. IFN-induced firefly luciferase activity was normalized to *Renilla* luciferase activity. The bars represent the mean fold induction of 3 experiments compared to the untreated negative control and error bars represent the standard deviations.

MARV VP40 contains a late domain (PPPY), positioned from residues 16–19, that mediates VP40 interaction with the cellular protein Tsg101, a component of the ESCRT I machinery, and contributes to its budding function [18]. To determine whether this late domain is critical for MARV VP40 inhibition of signaling, the 16-PPPY-19 motif was mutated to 16-AAAA-19 (M40-AAAA). Relative to wild-type EBOV VP40 or wild-type MARV VP40, M40-AAAA exhibited greatly reduced budding, in the form of virus-like particles (VLPs), from transfected 293T cells, despite comparable expression in the whole cell extracts (Fig. 8A). As expected, a separately expressed GFP was not released into the cell medium (Fig. 8A). When tested for its ability to suppress IFNα/β-induced signaling, the mutant suppressed STAT1 phosphorylation comparably to either LGTV NS5 or wild-type MARV VP40 (Fig. 8B). The mutant also suppressed IFNα/β-induced activation of the ISG54 promoter comparably to wild-type MARV VP40 (Fig. 8C). Therefore we conclude that the MARV VP40 late domain is not required for inhibition of signaling.

## Discussion

Previous studies have shown that both members of the filovirus family, MARV and EBOV, impair cellular responses to IFNs [30],[33],[34],[50]. While ZEBOV blocks the nuclear accumulation of tyrosine-phosphorylated STAT1 [33],[34], the present study demonstrates that MARV has evolved a different mechanism to counteract IFN signaling. We show that MARV inhibits the IFNα-induced tyrosine phosphorylation of not only STAT1 and STAT2 but also of the upstream kinases Jak1 and Tyk2. This inhibition prevents the IFN-induced nuclear accumulation of STAT1 and STAT2. Further, MARV infection inhibits the IFNγ-induced tyrosine phosphorylation of STAT1. The inhibition extends even beyond the IFNα/β and IFNγ signaling pathways to another Jak1 dependent signaling pathway, the IL-6 pathway, where the phosphorylation of STAT1 and STAT3 was inhibited. Significantly, the study also identifies a single MARV protein, the matrix protein VP40, sufficient to mediate these inhibitory effects, whereas ZEBOV-induced inhibition of IFN signaling is mediated by VP24 [33]. Emphasizing the specificity of the inhibitory function for MARV VP40, neither ZEBOV infection nor ZEBOV VP40 expression impairs Jak or STAT phosphorylation. Moreover, MARV VP24, including VP24s corresponding to the Musoke strain and the Angola strain, which caused an outbreak with a very high fatality rate [51], did not detectably inhibit IFNα/β-induced gene expression (Fig. 5B and data not shown). Musoke MARV VP24 was also unable to inhibit IFNα/β-, IFNγ- or IL-6-induced phosphorylation of Jaks or STATs.

The striking differences in the strategies employed by filoviruses to block IFN signaling may have been driven by the different evolutionary paths taken by Marburg and Ebola viruses. Bayesian analysis of genome sequence differences indicates that Ebola and Marburg viruses diverged from a common ancestor several thousands of years ago (S.T. Nichol, personal communication). Evolution in and adaptation to different host species might account for different immune evasion mechanisms. So far, there is only limited information available about the natural host spectrum of filoviruses. Various species of African fruit bats were found to be seropositive or RT-PCR-positive for EBOV [52],[53], however, as yet Ebola viruses have not been isolated from bats. In contrast, Towner and colleagues reported the successful isolation of MARV from the Egyptian fruit bat *Rousettus aegyptiacus* [54]. Since this bat species is also discussed as a potential reservoir for EBOV [53], it remains unclear if Marburg and Ebola viruses differ in their host tropism. Recently, the Asian EBOV species *Reston ebolavirus* (REBOV), which is thought to be non-pathogenic for humans, was isolated from pigs [55]. Phylogenetic analyses suggested that the REBOV clade has evolved separately from the African Ebola viruses [55]. Interestingly, REBOV VP24 was also shown to interfere with the nuclear translocation of STAT1 [34], indicating that the ability of VP24 to counteract IFN signaling was evolved among Ebola viruses prior to the separation of the African and Asian species. Notably, VP24 contributes to the host specificity of ZEBOV [24],[25]. Whether VP40 plays a similar role in MARV host tropism has yet to be determined; however, it is intriguing that a mouse-adapted MARV acquired amino acid changes in VP40 [56].

The effects of MARV infection and MARV VP40 expression on IFNα/β, IFNγ and IL-6 signaling mirror the impact of Jak1 knock-out on these pathways. In cells lacking Jak1, no STAT or Jak phosphorylation was observed upon IFNα/β or IFNγ treatment [5]. Similarly, the absence of Jak1 profoundly affects the IL-6 pathway as elimination of Jak1 was sufficient to fully abrogate any detectable phospho-STAT1 and greatly reduce phospho-STAT3 following IL-6 addition [7],[8]. Interestingly, MARV infection and individual expression of MARV VP40 closely mirror this phenotype, where following IL-6 addition, phospho-STAT1 was undetectable but residual phospho-STAT3 was present (Fig. 6). Further studies will reveal to what extent the observed residual STAT3 phosphorylation may mediate IL-6 signaling.

Our data are consistent with a model in which MARV VP40 targets Jak1 function, either directly or indirectly, although the possibility remains that MARV VP40 can also impair signaling of other Jak family kinases. A possible indirect mechanism of the observed inhibition could be a modulating effect of MARV VP40 on PTPs targeting Jak kinases. Recently, it has been reported that transgenic mice with reduced expression of the PTP CD45 were protected against lethal EBOV infection [56]. Interestingly, CD45 acts as a negative regulator of Jak1 in cells of hematopoietic origin [57]. However, our data suggest that PTPs are not involved in MARV-mediated inhibition of Jak1 signaling in cells of non-hematopoietic origin. Therefore, it is of interest to further extend those studies and to analyze Jak/STAT signaling in human hematopoietic cells in the context of MARV and EBOV infection.

The observed inhibitory effects of MARV VP40 on both IFNα/β-induced gene expression and the antiviral effects of IFNβ may explain the capacity of MARV to prevent cellular responses to exogenously-added IFNα [30]. In this respect, MARV VP40 appears to serve the same purpose as the EBOV VP24 proteins which also counteract IFNα/β signaling. It is likely that counteracting IFNα/β signaling has a significant impact on viral pathogenesis *in vivo*, because, despite the presence of viral VP35 proteins that suppress IFNα/β production [31],[58],[59],[60], filovirus replication *in vivo* results in significant IFNα production [61]. The presence of IFNα/β signaling inhibitors likely also contributes to the relative insensitivity of filoviruses to IFNα/β as an antiviral therapy [50]. IFNγ also has antiviral properties [62], however, suppression of IFNγ signaling may also modulate adaptive immune responses to infection. For example, human cytomegalovirus down-regulates Jak1 expression in a proteasome-dependent manner, and although a specific viral gene product that mediates this effect has not been defined, this function prevents the IFNγ-induced upregulation of MHC class II on infected cells [63]. Another viral protein that interacts with Jak1 and blocks the type I IFN signaling pathway is the measles virus V protein, but the consequence of this function for adaptive immunity has not been defined [64]. The possible impact of MARV infection and MARV VP40 expression on other cytokine signaling pathways involving Jak1 remains to be defined. Given the prominent role of Jak1 in numerous pathways, the impact of MARV VP40 on cytokine signaling may be quite broad.

Filovirus VP40 proteins are matrix proteins sufficient to drive budding of virus-like particles, and they are thought to be the driving force for the budding of infectious virus [11],[13],[18],[65],[66],[67]. The finding that MARV VP40 also serves as an inhibitor of IFN signaling is surprising and novel. Another example of a negative-strand RNA virus matrix protein that inhibits IFN responses is the vesicular stomatitis virus (VSV) matrix protein (M). VSV M inhibits innate immune responses, including IFNβ production, by a mechanism different from MARV VP40, inhibiting host cell transcription as well as nucleo-cytoplasmic transport of cellular mRNAs [68],[69],[70],[71].

Host factors that interact with filovirus VP40 proteins have been described [14],[18],[65],[72],[73]. The most fully characterized interactions occur via the VP40 late domain which facilitates budding and release of virus particles. ZEBOV VP40 possesses two late domains, a PTAP motif and an overlapping PXXP motif [11],[65]. These mediate interaction with Tsg101, Nedd4, and Rsp5 [14],[65]. MARV VP40 possesses a single PPPY motif that allows interaction with Tsg101 [18]. To address the potential role of these well-characterized motifs in MARV VP40 inhibition of Jak/STAT signaling, a 16-PPPY-19 to 16-AAAA-19 mutant MARV VP40 was generated. As previously described, this mutation severely impaired MARV VP40 budding (Fig. 8A) [18]. Yet this mutation had no detectable impact on MARV VP40 inhibition of IFNα/β signaling (Fig. 8B and C). Therefore, the late domain is dispensable for the IFN signaling function of VP40, and the budding and signaling functions of MARV VP40 appear to be separable. Of note, IFN-induced cellular inhibitors of filovirus VP40 budding have recently been described. These include the IFN stimulated ISG15 and tetherin [26],[74],[75]. ISG15 is an IFN-induced protein which inhibits budding of EBOV VP40. ISG15 inhibits the ubiquitin ligase Nedd4, which interacts with EBOV VP40 through the PPXY motif to promote VP40 ubiquitination and budding [65],[75],[76]. Tetherin is constitutively-expressed in some cell types but is also IFN-inducible. Its expression can prevent release of VLPs produced following expression of EBOV or MARV VP40 [26],[74]. Co-expression of EBOV GP has been shown capable of counteracting this antiviral function [26]. Whether MARV GP can also inhibit tetherin has not yet been addressed; however, because MARV VP40 can inhibit IFN signaling, it appears to have a built-in capacity to resist IFN-induced mechanisms that target viral budding.

This study has identified an important difference in the biology of MARV and EBOV, defined a novel function for the MARV VP40 matrix protein and suggests that MARV may inhibit multiple Jak1-dependent cytokine signaling pathways. Future studies will determine whether the different means by which EBOV and MARV counteract cell signaling pathways result in significant differences in the pathologenesis of these viruses. Determining the molecular mechanisms by which MARV VP40 blocks signaling may facilitate development of new anti-MARV therapies.

## Materials and Methods

### Cell lines and viruses

293T, Vero E6, Vero (ATCC, Manassas, VA) and Huh-7 (kindly provided by Dr. DiFeo, Mount Sinai School of Medicine) cells were maintained in Dulbecco's modified Eagle medium (DMEM) supplemented with 10% fetal bovine serum and 10 mM HEPES pH 7.5 or in DMEM supplemented with penicillin (50 units/ml), streptomycin (50 mg/ml) and 10% fetal bovine serum. HUVECs were maintained in F-12K medium (ATCC) supplemented with 0.1 mg/ml heparin (Sigma-Aldrich, St. Louis, MO), 0.03 mg/ml endothelial cell growth supplement (ECGS) (Sigma-Aldrich), and 10% fetal bovine serum (HyClone). A previously-described Newcastle disease virus engineered to express green fluorescence protein (NDV-GFP) was propagated in 10-day-old embryonated chicken eggs [77]. ZEBOV strain Mayinga and MARV strain Musoke were grown and propagated as described previously [30]. All work with infectious filoviruses was performed under biosafety level 4 conditions at the Institute of Virology, Philipps University of Marburg, Marburg, Germany.

### Plasmids

PCR products corresponding to FLAG-tagged, HA-tagged or untagged viral proteins of EBOV (Accession # NC002549) and MARV (Accession # NC001608) were cloned into the pCAGGS or pcDNA3.1 expression vectors [78]. The Nipah Virus W (NiV W) protein expression plasmid was previously described [77]. The expression plasmid for V5-tagged Langat Virus NS5 was previously described [46]. Human Jak1 (Accession # BAE02826) and Tyk2 (Accession # NP_003322) were RT-PCR amplified from RNA isolated from 293T cells and cloned with an HA tag into the pCAGGS vector. For the generation of the late domain mutants, site directed mutagenesis was performed using the QuickChange XL II kit (Stratagene, La Jolla, CA). A Flag-tagged Rabies P expression plasmid in a pCR3 background was kindly provided by Drs. Conzelmann and Brzozka (Ludwig Maximilian University, Munich, Germany).

### Transfections

293T cells were transfected using Lipofectamine 2000 (LF2K) at a ratio 1∶1 with plasmid DNA (µg DNA: µL LF2K). Vero cells were transfected using LF2K at a ratio 1∶2. Huh-7 cells were transfected using LF2K at a ratio 1∶2.75. HUVEC cells were electroporated using the AMAXA nucleofector II, nucleofection program V-001 and solution V according to the manufacturer's directions (Lonza, Walkersville, MD). Cells were lysed with an IGEPAL lysis buffer (50 mM Tris [pH 8.0], 280 mM NaCl, 0.5% IGEPAL, 0.2 mM EDTA, 2 mM EGTA, 10% glycerol, 1 mM dithiothreitol (DTT) supplemented with protease inhibitor cocktail (Roche) and 0.1 mM Na_3_VO_4_) [79] for 30 min on ice and spun at 13kRPM on a refrigerated tabletop centrifuge for 1 minute.

### Cytokines

Universal type I IFN (a consensus IFNα/β), human IFNβ and human IFNγ (PBL, Piscataway, NJ) were used at 1000 IU/ml unless otherwise specified for 30 min in RPMI (GIBCO) or phosphate buffered saline (PBS) supplemented with 0.3% BSA. Human IFNα-2b (Essex Pharma, Kenilworth, NJ) diluted in PBS supplemented with 0.1% BSA was used at 1000–2000 IU/ml unless otherwise specified. Human TNFα (Peprotech, Rocky Hill, NJ) was used at 50 ng/ml for 24 hours in HUVEC culture medium as described above. Human IL-6 (Peprotech, Rocky Hill, NJ) was used at 50 ng/ml in RPMI supplemented with 0.3% BSA.

### Inhibition of IFNβ-induced antiviral state

4×10^5^ Vero cells per well were cultured in 24 well plates and transfected with 1 µg of each plasmid encoding viral proteins. At 24 hours post-transfection cells were treated with IFNβ (1000 IU/ml) for 24 hours. Then cells were infected with 5 hemagglutinating units of NDV-GFP virus in a volume of 200 µl of 0.3% BSA in PBS for 1 h, washed twice and replaced with DMEM supplemented with 10% FBS. GFP expression was visualized at 16 hours post-infection with a fluorescence microscope.

### Reporter gene assays

293T cells (5×10^5^) or Huh-7 (3×10^5^) were transfected with 0.5 µg of a construct having an IFN-stimulated gene 54 promoter driving expression of a chloramphenicol acetyltransferase (CAT) reporter gene (pISG54-CAT), 0.1 µg of a constitutively expressing *Renilla* luciferase reporter construct (pCAGGS-luc), and the indicated amounts of the expression plasmids. Twenty-four hours post-transfection, cells were washed and treated with IFN (as described above). Sixteen hours post-IFN treatment, cells were harvested using reporter lysis buffer (Promega, Madison, WI) and analyzed for CAT and luciferase activities by standard methods. CAT activity was quantified by using a PhosphorImager and normalized to the luciferase activity. Alternatively, an ISG54-firefly luciferase reporter plasmid (pISG54-Luc) (0.3 µg) reporter was used, and a dual luciferase reporter (DLR) assay was performed according to the manufacturer's guidelines (Promega). For IFNγ-dependent gene expression, a reporter having 3 copies of the gamma activated sequence driving the expression of firefly luciferase (GAS-Luc) (0.3 µg) was transfected with 0.1 µg of a constitutively expressing luciferase reporter construct (pCAGGS-luc), and the indicated amounts of the expression plasmids. Twenty-four hours post-transfection, cells were washed and treated with IFNγ (as described above). Sixteen hours post-IFN treatment cells were harvested and analyzed using a DLR assay (Promega). Assays were performed in triplicate and p-values were calculated by a two-tailed Student's t-test for unpaired samples using the software GraphPad Prism (GraphPad Software, Inc.).

### Western blot analysis of transfected cells and ELISAs

For the detection of the overexpressed viral proteins, the anti-V5 (Invitrogen), anti-HA and anti-Flag M2 (Sigma) antibodies were used at a 1∶5000 dilution in 1% non-fat dry milk in Tris-buffered saline (TBS; 20 mM Tris-HCl, pH 7.4; 150 mM NaCl). As a loading control, anti beta-tubulin (Sigma) antibody was used at a 1∶10,000 dilution in 1% non-fat dry milk in TBS. Anti-GFP was used at a 1∶10,000 dilution in 1% non-fat dry milk in TBS (Clontech, Mountain View, CA). Phosphorylated STAT1 was detected with a phospho-tyrosine specific antibody recognizing phospho-Y701 (BD Transduction Laboratories, San Jose, CA), and total levels of STAT1 with an antibody recognizing the STAT1 C-terminus (BD Transduction Laboratories) diluted to 1∶1000 and 1∶500, respectively, in 1% non-fat dry milk in TBS. STAT2 and its phosphorylated form (pY689) were detected with polyclonal antibodies (Santa Cruz Biotechnology, Santa Cruz, CA and Upstate, Lake Placid, NY respectively) diluted 1∶500 in 1% non-fat dry milk in TBS. STAT3, pY705-STAT3, Tyk2, pY1054/1055-Tyk2, pY1022/1023-Jak1, pY1007/1008-Jak2 (Cell Signaling, Beverly, MA), Jak1 (BD Transduction Laboratories) and Jak2 (Millipore, Billerica, MA) were used at a 1∶500 dilution in TBS, 0.1% Tween and 5% BSA.

For the detection of IP-10 and IL-8, supernatants of transfected HUVECs treated with 100 IU/ml human IFNγ or 50 ng/ml TNFα for 24 hours were collected and diluted 1∶100 and 1∶1000, respectively, in PBS supplemented with 5% fetal bovine serum. The BD OptEIA Human IP-10 and Human IL-8 kits were used (BD Biosciences, Franklin Lakes, NJ).

### Western blot analysis of filovirus-infected cells

Huh-7 cells grown in six-well plates to approximately 50% confluence were infected with ZEBOV or MARV at an MOI of 5. At 24 hours p.i., cells were left untreated or treated with IFNα-2b (concentrations indicated in the figure legends), 10 IU/ml IFNγ or 50 ng/ml IL-6 for 20 or 30 min, respectively. Where indicated, filovirus-infected cells were treated with the phosphatase inhibitors sodium orthovanadate (Sigma; 167 µM, 4 h) or PTPIB-Inhibitor (Merck; 33 µM, overnight; addition of fresh inhibitor the next morning for 40 min), or DMSO (Sigma) prior to IFN treatment. These conditions were shown to be sufficient to block Tyk2 dephosphorylation in non-infected cells treated with IFNα for 60 minutes in the presence of phosphatase inhibitors (data not shown). Thereafter, cells were washed twice with PBS and scraped into 2× protein loading buffer (114 mM Tris-HCl, pH 6.8; 2.5% SDS; 125 mM dithiothreitol; 25% glycerol; 0.25% bromphenol blue). Cell lysates were transferred to fresh tubes, boiled for 2.5 to 10 min and subjected to SDS-polyacrylamide gel electrophoresis. Proteins were blotted onto polyvinylidene difluoride membranes, and the membranes were blocked in 5% non-fat dry milk in TBS containing 0.1% Tween 20 for 1 hour at room temperature, followed by an incubation step with the appropriate primary antibody in TBS supplemented with 5% bovine serum albumin and 0.1% Tween 20 overnight at 4°C. To detect endogenous cellular proteins, the following antibodies were used: rabbit anti-STAT1-phospho Tyr 701 (CST; dilution 1∶3000), rabbit anti-STAT1-total (BD transduction; dilution 1∶3000), rabbit anti-STAT2-phospho Tyr 689 (Biomol; dilution 1∶1000), rabbit anti-STAT2-total (Imgenex; dilution 1∶1000), rabbit-anti-STAT3-phospho Tyr705 (CST; dilution 1∶500), rabbit-anti-STAT3-total (Santa Cruz; dilution 1∶500), rabbit-anti-Tyk2-phospho Tyr1054/1055 (CST; dilution 1∶3000), rabbit-anti-Tyk2-total (Santa Cruz, dilution 1∶3000), rabbit-anti-Jak1-phospho Tyr1022/1023 (Biomol; dilution 1∶1000) and rabbit-anti-Jak1-total (Santa Cruz; dilution 1∶1000). Horseradish peroxidase-conjugated secondary antibodies (Dianova) were used and visualized by using either the chemiluminescence substrate SuperSignal West Dura Extended Duration or SuperSignal West Femto Maximum Sensitivity (Pierce) according to the manufacturer's instructions. To verify virus infection, infected and IFN-treated cells grown on glass coverslips were subjected to immunofluorescence analysis using virus-specific antibodies as described below.

### Immunofluorescence analysis

Huh-7 cells grown on glass coverslips were infected with ZEBOV or MARV at an MOI of 5 or left uninfected. At 24 hours p.i., cells were washed twice with PBS and inactivated by treatment with 4% paraformaldehyde for 24 hours. Cells were then permeabilized with a mixture of acetone and methanol (1∶1, vol/vol) for 5 min at −20°C and treated with 0.1 M glycine. As primary antibodies, a rabbit antiserum directed against the nucleocapsid complex of MARV (1∶100) and a goat antiserum directed against ZEBOV (1∶500) (kindly provided by Dr. Becker, Philipps University of Marburg, Marburg, Germany) were used. To detect endogenous STAT1 or STAT2 proteins in filovirus-infected cells, the cells were fixed in 4% paraformaldehyde as described above, washed with 50 mM NH_4_Cl in PBS and permeabilized with 0.5% Triton X-100 in PBS. After incubation with primary antibodies (rabbit anti-STAT1 or rabbit anti-STAT2 (Santa Cruz; dilution 1∶100) along with filovirus-specific antibodies), the specimens were washed with PBS and incubated with fluorescence-labeled secondary antibodies.

To analyze the intracellular localization of endogenous STAT2 in cells expressing individual viral proteins, Huh-7 cells were transfected with 2 µg of plasmid DNA encoding MARV or ZEBOV VP40, VP35, or VP24 using FuGene 6 (Roche) according to the manufacturer's instructions. VP24 proteins and ZEBOV VP35 were tagged with an HA epitope. As a control cells were transfected with 2 µg pCR3 Flag-tagged rabies virus P. Immunofluorescence analysis was performed by using antibodies directed against STAT2, MARV VP35 (mouse; 1∶100), MARV VP40 (mouse; 1∶100), ZEBOV VP40 (mouse; 1;100), Flag- (Sigma; dilution 1∶700) or HA-tags (Sigma; dilution 1∶1000).

### VLP budding assay

293T cells were transfected with 2 µg of expression plasmid. At 48 hours post-transfection, cell culture supernatants were clarified by centrifugation at 200×*g* for 5 min and pelleted through a 20% sucrose cushion in NTE buffer (100 mM NaCl, 10 mM Tris [pH 7.5], 1 mM EDTA [pH 8.0]) at 160,000×*g* for 2 hours at 4°C. Supernatants were aspirated and the pellets containing the virus-like particles (VLPs) were resuspended in NTE buffer. Cells were washed with PBS and lysed in radioimmunoprecipitation assay buffer (RIPA) (50 mM Tris [pH 7.4], 150 mM NaCl, 0.1% sodium dodecyl sulfate [SDS], 0.5% deoxycholate, 1% NP-40) supplemented with protease inhibitor cocktail (Roche). VLPs and lysates were analyzed by SDS-PAGE and visualized by western blotting, as described [80].
